# Rooibos Flavonoids, Aspalathin, Isoorientin, and Orientin Ameliorate Antimycin A-Induced Mitochondrial Dysfunction by Improving Mitochondrial Bioenergetics in Cultured Skeletal Muscle Cells

**DOI:** 10.3390/molecules26206289

**Published:** 2021-10-18

**Authors:** Sinenhlanhla X. H. Mthembu, Christo J. F. Muller, Phiwayinkosi V. Dludla, Evelyn Madoroba, Abidemi P. Kappo, Sithandiwe E. Mazibuko-Mbeje

**Affiliations:** 1 Biomedical Research and Innovation Platform, South African Medical Research Council, Tygerberg, Cape Town 7505, South Africa; sinenhlanhla.mthembu@mrc.ac.za (S.X.H.M.); christo.muller@mrc.ac.za (C.J.F.M.); phiwayinkosi.dludla@mrc.ac.za (P.V.D.); 2 Department of Biochemistry and Microbiology, University of Zululand, KwaDlangezwa 3886, South Africa; MadorobaE@unizulu.ac.za; 3 Department of Biochemistry, Faculty of Natural and Agricultural Sciences, Mafikeng Campus, North West University, Private Bag X 2046, Mmabatho 2735, South Africa; 4 Division of Medical Physiology, Faculty of Health Sciences, Kingsway Campus, Stellenbosch University, Tygerberg, Cape Town 7505, South Africa; 5 Department of Biochemistry, Faculty of Science, University of Johannesburg, Auckland Park, Johannesburg 2006, South Africa; akappo@uj.ac.za

**Keywords:** antimycin A, mitochondrial dysfunction, skeletal muscle, isoorientin, orientin, aspalathin, bioenergetics, biogenesis

## Abstract

The current study investigated the physiological effects of flavonoids found in daily consumed rooibos tea, aspalathin, isoorientin, and orientin on improving processes involved in mitochondrial function in C2C12 myotubes. To achieve this, C2C12 myotubes were exposed to a mitochondrial channel blocker, antimycin A (6.25 µM), for 12 h to induce mitochondrial dysfunction. Thereafter, cells were treated with aspalathin, isoorientin, and orientin (10 µM) for 4 h, while metformin (1 µM) and insulin (1 µM) were used as comparators. Relevant bioassays and real-time PCR were conducted to assess the impact of treatment compounds on some markers of mitochondrial function. Our results showed that antimycin A induced alterations in the mitochondrial respiration process and mRNA levels of genes involved in energy production. In fact, aspalathin, isoorientin, and orientin reversed such effects leading to the reduced production of intracellular reactive oxygen species. These flavonoids further enhanced the expression of genes involved in mitochondrial function, such as *Ucp 2*, *Complex 1/3*, *Sirt 1*, *Nrf 1*, and *Tfam*. Overall, the current study showed that dietary flavonoids, aspalathin, isoorientin, and orientin, have the potential to be as effective as established pharmacological drugs such as metformin and insulin in protecting against mitochondrial dysfunction in a preclinical setting; however, such information should be confirmed in well-established in vivo disease models.

## 1. Introduction

Skeletal muscle insulin resistance is considered one of the primary defects in type 2 diabetes (T2D) [1,2], but the precise mechanism(s) that leads to this phenomenon has not been fully elucidated. The prevailing hypothesis suggests that impairments in mitochondrial oxidative capacity might be an underlying defect that causes insulin resistance, thereby contributing to the pathogenesis of T2D [3]. In experimental models of metabolic disease, genetic dysregulations linked with reduced mitochondrial DNA content (mDNA) and downregulation of nuclear respiratory factor 1 (NRF 1), as well as mitochondrial transcription factor (TFAM), are persistent with impaired processes of cellular bioenergetics and biogenesis [4,5]. In fact, excessive production of intracellular reactive oxygen species (ROS) has occurred consistent with dysregulations in the mitochondrial respiration process [6,7], exacerbation of an undesired pro-inflammatory response [8], and unbalanced energy expenditure (this mainly includes the ratio of AMP:ATP production) [9]. Therefore, studies that can enhance our understanding of the implications of mitochondrial function in target tissues such as the skeletal muscle are of crucial importance for the development of novel drugs or therapeutic strategies to improve cellular function and slowing down the progression of conditions such as T2D.

**Figure 1:**
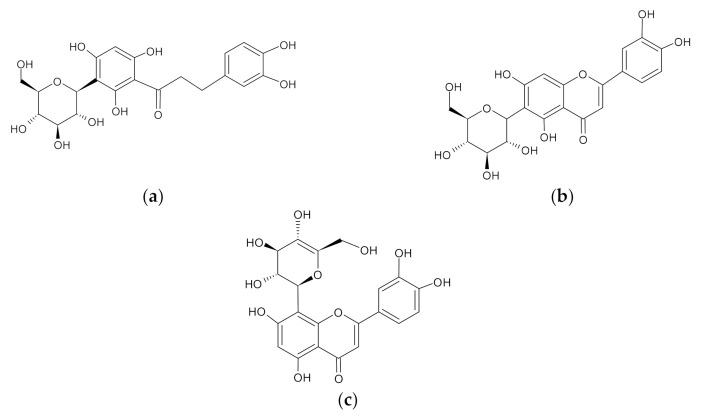
Chemical structures of aspalathin (**a**), isoorientin (**b**), and orientin (**c**).

Recently, researchers have reported that regular intake of polyphenols has been linked with the reduced risk of life-threatening diseases, including diabetes and other metabolic complications [10,11,12]. In fact, natural compounds such as genipin, resveratrol, quercetin, ursolic acid, and cinnamon have been reported to enhance insulin sensitivity and improve mitochondrial function in various preclinical models of metabolic disease [12,13,14,15,16,17]. Previous studies have shown that rooibos-rich polyphenolic compounds such as aspalathin and nothofagin can attenuate inflammation, oxidative stress, and insulin resistance in vitro and in vivo [11,18,19,20]. Rooibos is an indigenous South African herbal tea made from the leaves of *Aspalathus linearis* [21]. This plant has gained popularity lately for its envisaged health properties such as antidiabetic [22], anti-obesity [23], cardio-protective [24], anti-cancer [25], wound healing [26], and other protective properties against metabolic complications [27]. Rooibos also contains essential flavones (Figure 1), including orientin and isoorientin, which are known to be the oxidative products of nothofagin and aspalathin [28].

According to Joubert and de Beer, 2011, [29], aspalathin is the most abundant polyphenol in rooibos, followed by isoorientin and its isoform, orientin. Notably, even though more attention has been given to aspalathin, there is increasing evidence on the beneficial properties of isoorientin and orientin. Briefly, it has been reported that isoorientin has anti-inflammatory properties and the ability to ameliorate mitochondrial ROS production [30,31]. Moreover, in our laboratory, we have reported that isoorientin reduces lipid accumulation by regulating energy metabolism and the expression of genes involved in the browning of fat, such as peroxisome proliferator-activated receptor gamma/alpha (PPARg/α) and uncoupling protein 1 (UCP 1) in vitro [32]. A similar effect has been reported by others, showing that orientin attenuates chemically induced inflammation by inactivating nuclear factor kappa light chain enhancer of activated β cells (NF-kB) and mitogen-activated protein kinase (MAPK) pathways [33]. However, the effect of rooibos flavonoids such as aspalathin, isoorientin, and orientin on mitochondrial function, especially the process of bioenergetics within the skeletal muscle, has not been fully described. Therefore, this study, for the first time, reports on the ameliorative effects of aspalathin, isoorientin, and orientin against some dysregulations in mitochondrial function in cultured skeletal muscle cells.

## 2. Results

### 2.1. Aspalathin, Isoorientin, and Orientin Reduced ROS Production and Increased the Expression of Some Antioxidant Genes

**Figure 2:**
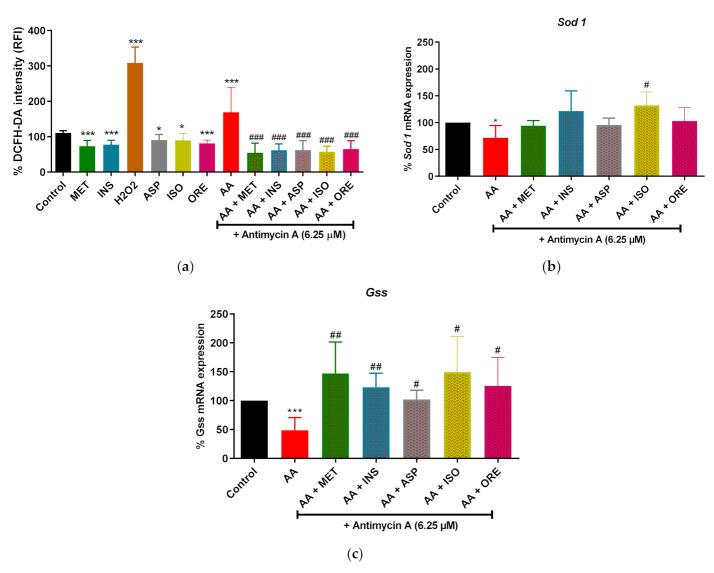
The impact of aspalathin, isoorientin, and orientin on the production of reactive oxygen species (ROS) (**a**) and the expression of antioxidants genes such as superoxide dismutase 1 (*Sod1*, (**b**)) and glutathione synthetase (*Gss*, (**c**)) in cultured C2C12 myotubes following the exposure to antimycin A. Briefly, C2C12 cells were treated with antimycin A (6.25 µM) for 12 h to induce mitochondrial dysfunction. Thereafter, cells were treated with aspalathin (Asp), isoorientin (Iso), orientin (Ore) (10 µM), and comparative control metformin (Met) (1 µM) for 4 h. Insulin (Ins) (1 µM) and H_2_O_2_ (1000 µM) (ROS positive control) were added for 30 min. Results are expressed as mean ± SD of three independent experiments. * *p* < 0.05, *** *p* < 0.001 versus normal control; ^#^ *p* < 0.05, ^##^ *p* < 0.01, ^###^ *p* < 0.001 versus antimycin A control. Dichlorofluoresceine-diacetate (DCFH-DA) green fluorescence stain (intensity) was used as a measurement of ROS production.

Firstly, we evaluated the effect of aspalathin, isoorientin, and orientin on normal physiological conditions. Our data showed that metformin and insulin, which were used as comparators, significantly reduced the ROS production in normal cells (*p* < 0.001), as shown in Figure 2a. Alternatively, exposing the cells to a high concentration of H_2_O_2_, which was used as a positive control for ROS, resulted in a significant increase in ROS production (*p* < 0.001) (Figure 2a). Whereas aspalathin, isoorientin, and orientin showed a significant decrease in ROS production under normal conditions (*p* < 0.05, *p* < 0.01 and *p* < 0.001, respectively) (Figure 2a). Moreover, cells that were exposed to antimycin A (6.25 µM) showed a significant increase in ROS production (*p* < 0.001) (Figure 2a). However, adding insulin, metformin, aspalathin, isoorientin and orientin significantly reduced the ROS production in cells treated with Antimycin A (*p* < 0.001) (Figure 2a) In addition, antimycin A markedly reduced the expression of antioxidant genes such as *Sod 1* and *Gss* (*p* < 0.05, and *p* < 0.001, respectively) (Figure 2b,c). Interestingly this effect was reversed by metformin, insulin, aspalathin, isoorientin, and orientin. Notably, only isoorientin showed an enhanced effect in increasing the expression of *Sod 1* (*p* < 0.05) following exposure to antimycin A (Figure 2b).

### 2.2. Aspalathin, Isoorientin, and Orientin Enhance the Parameters of Mitochondrial Respiration and Glycolysis following Exposure to Antimycin A in Cultured Skeletal Muscle Cells

**Figure 3:**
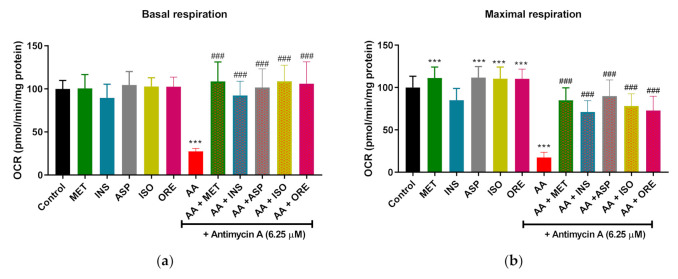

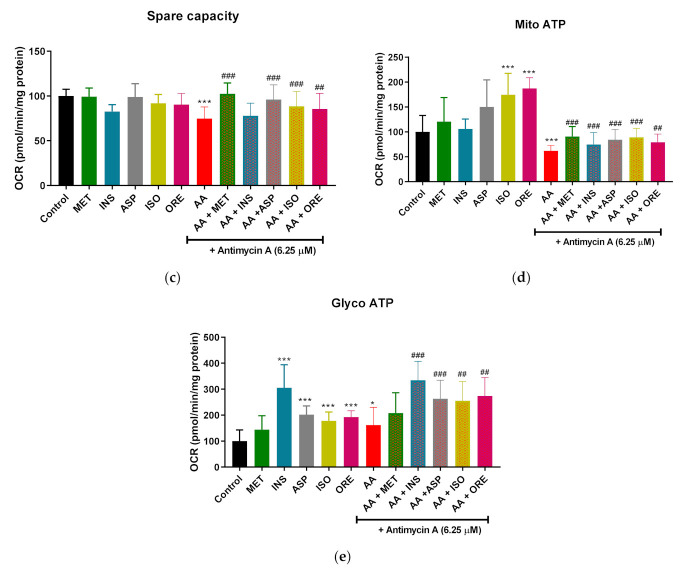
The effect of aspalathin, isoorientin, and orientin on oxygen consumption rate (OCR) and real-time ATP production in C2C12 skeletal muscle cells. Briefly, figure panels (**a**–**e**) represent basal respiration, maximal respiration, spare capacity, mitochondrial ATP, and glycolytic ATP, respectively. C2C12 were treated with antimycin A (6.25 µM) for 12 h followed by treatment with aspalathin, isoorientin, orientin (10 µM), and comparative control metformin (1 µM) for 4 h. Insulin (1 µM) for 30 min. Results are expressed as mean ± SD of three independent experiments. * *p* < 0.05, *** *p* < 0.001 versus normal control; ^##^ *p* < 0.01, ^###^ *p* < 0.001 versus antimycin A control.

Mitochondrial respiration and real-time ATP production were assessed using seahorse Mito stress and real-time ATP assays. The levels of oxygen consumption rate (OCR) were measured. Under normal physiological conditions, metformin showed a significant increased on maximal respiration (*p* < 0.001) while insulin increased glycolytic ATP (*p* < 0.001) (Figure 3e). Moreover, Aspalathin, isoorientin, and orientin significantly increased maximal respiration, mitochondrial (only isoorientin and orientin) and glycolytic ATP (*p* < 0.001) under physiological conditions (Figure 3b,d,e). Looking at our results, it was clear that antimycin A (6.25 µM) significantly reduced basal, maximal respiration, and spare capacity (*p* < 0.001) (Figure 3a–c). However, this effect was reversed by metformin and insulin (*p* < 0.001) as a comparative control except in the spare capacity (Figure 3a–c). Interestingly, co-treating of antimycin A with rooibos flavonoids such as aspalathin, isoorientin, and orientin significantly enhanced basal, maximal respiration, and spare capacity (*p* < 0.001) (Figure 3a,c). We further investigated the effect of these flavonoids on ATP synthesis by measuring the real-time ATP reproduction. Consistent with suppression of mitochondrial respiration, antimycin A significantly decreased the rate of mitochondrial ATP (*p* < 0.01) (Figure 3d). This effect was significantly reversed by metformin, insulin, aspalathin, isoorientin, and orientin (*p* < 0.001, *p* < 0.01, respectively). Furthermore, cells treated with antimycin A showed an increase in glycolytic ATP (*p* < 0.001) (Figure 3e). Even further increase in glycolytic ATP production was observed in the cells that were co-treated with antimycin A and insulin, aspalathin, isoorientin, or orientin (*p* < 0.001, *p* < 0.001, *p* < 0.01, and *p* < 0.01, respectively) compared to antimycin A control.

### 2.3. Aspalathin, Isoorientin and Orientin Modulates the mRNA Expression of Genes Involved in Mitochondrial Bioenergetics following Exposure to Antimycin A in Cultured Skeletal Muscle Cells

**Figure 4:**
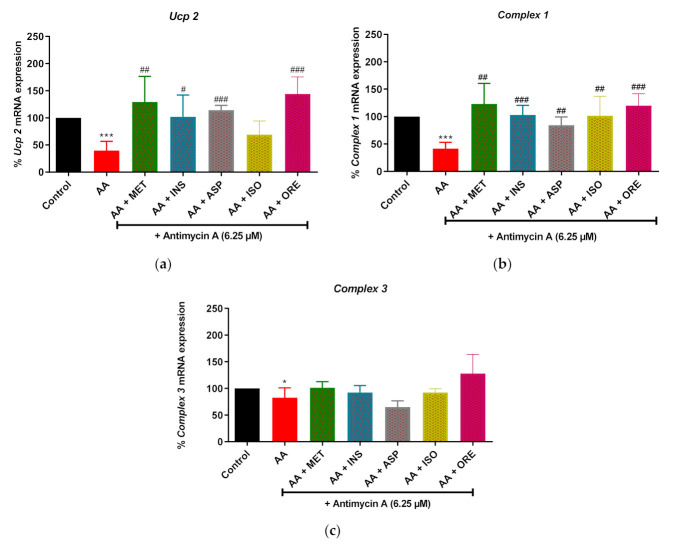
The effect of aspalathin, isoorientin, and orientin on the expression of mitochondrial bioenergetics genes; uncoupling protein 2 (*Ucp 2*) (**a**), *Complex 1* (**b**), and *Complex 3* (**c**) in C2C12 cells exposed to antimycin A. Cells were exposed to 6.25 µM antimycin A for 12 h, followed by treatment with aspalathin, isoorientin, orientin (10 µM) and comparative control metformin (1 µM) for 4 h. Insulin (1 µM) for 30 min. Results are expressed as mean ± SD of three independent experiments. * *p* < 0.05, *** *p* < 0.001 versus normal control; ^#^ *p* < 0.05, ^##^ *p* < 0.01, ^###^ *p* < 0.001 versus antimycin A control.

Consistent with the reduced mitochondrial respiration (Figure 3), antimycin A also significantly reduced the expression of mRNA levels of genes involved in mitochondrial bioenergetics, *Ucp 2*, *Complex 1*, and *Complex 3* (*p* < 0.001, *p* < 0.001, and *p* < 0.050, respectively) (Figure 4). Metformin and insulin, as comparators, were able to improve the expression of *Ucp 2* (*p* < 0.01 and *p* < 0.05, respectively), *Complex 1* (*p* < 0.01 and *p* < 0.001, respectively), and *Complex 3* (no significance observed), (Figure 4). Interestingly, aspalathin, isoorientin, and orientin enhanced the expression of *Ucp 2* (*p* < 0.001, but isoorientin was not significant), and *Complex 1* (*p* < 0.01, *p* < 0.001 and *p* < 0.01, respectively), in cultured skeletal muscle cells exposed to antimycin A (Figure 4).

### 2.4. Aspalathin, Isoorientin, and Orientin Enhanced the mRNA Expression of Genes of the Markers of Mitochondrial Biogenesis in Cultured Skeletal Muscle Cells

**Figure 5:**
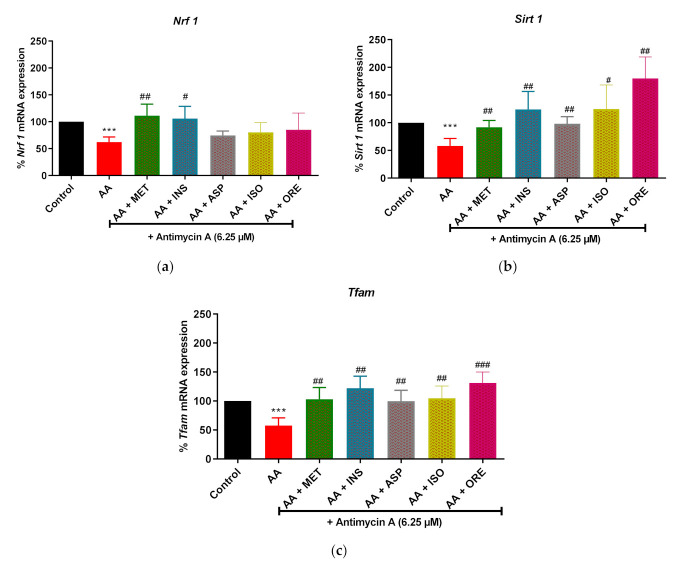
The effect of isoorientin and orientin on the expression of mitochondrial biogenesis genes; nuclear respiratory factor 1 (*Nrf 1*; (**a**)), Sirtuin 1 (*Sirt 1*; (**b**)), and mitochondrial transcription factor A (*Tfam*; (**c**)) in C2C12 cells exposed to antimycin A. Cells were exposed to 6.25 µM antimycin A for 12 h, followed by treatment with aspalathin, isoorientin, orientin (10 µM) and comparative control metformin (1 µM) for 4 h. Insulin (1 µM) for 30 min. Results are expressed as mean ± SD of three independent experiments. *** *p* < 0.001 versus normal control; ^#^ *p* < 0.05, ^##^ *p* < 0.01, ^###^ *p* < 0.001 versus antimycin A control.

We next assessed the effect of aspalathin, isoorientin, and orientin on antimycin A-induced alterations in the mRNA expression levels of some important genes involved in mitochondrial biogenesis. In this study, the expression of nuclear respiratory factor −1 *(Nrf 1*) (*p* < 0.001), *Sirt 1* (*p* < 0.001), *Tfam* (*p* < 0.001), were significantly decreased in skeletal muscle cells treated with antimycin A (Figure 5). Metformin and insulin, as comparators, were able to improve the expression of these genes, as depicted by enhanced mRNA levels of *Nrf 1* (*p* < 0.01 and *p* < 0.001, respectively), *Sirt 1* (*p* < 0.05 and *p* < 0.001, respectively), and *Tfam* (*p* < 0.05 and *p* < 0.001, respectively) (Figure 5). Although no significance was observed with mRNA levels of *Nrf 1*, the treatment compounds aspalathin, isoorientin and orientin did enhance the expression of *Sirt 1* (*p* < 0.05, *p* < 0.01 and *p* < 0.01, respectively) and *Tfam* (*p* < 0.01, *p* < 0.001 and *p* < 0.01, respectively), in cultured skeletal muscle cells exposed to antimycin A (Figure 5).

## 3. Discussion

Initially, we evaluated the effects of rooibos flavonoids, aspalathin, isoorientin, and orientin under physiological conditions. Our current study demonstrated that these rooibos flavonoids did not induce any major detrimental effects but slightly improved mitochondrial respiration while reducing the production of ROS in C2C12 skeletal muscle cells under normal conditions (in cells not exposed to antimycin A). Next, we investigated the effect of these flavonoids on markers of mitochondrial function in response to antimycin A (6.25 µM) exposure. This was relevant since antimycin A exposure in cultured cells is becoming a common experimental model to induce mitochondrial dysfunction because of its ability to block the activity of *Complex 3*, resulting in the collapse of the mitochondrial oxidative capacity, leading to elevated overproduction of ROS [34,35]. Indeed, antimycin A exposure was able to elevate ROS production when compared to the control. This consequently was concurrent with the capacity of this channel blocker to decrease mitochondrial respiration by shutting down the electron transport chain as demonstrated through the reduced generation of ATP production, basal and maximal respiration, as well as spare capacity. Moreover, antimycin A increased the glycolytic ATP production and further down-regulated the expression of genes involved in mitochondrial bioenergetics (*Ucp 2*, *Complex 1* and *3*) and biogenesis (*Nrf 1*, *Sirt 1*, and *Tfam*). These results were consistent with the data reported by Mazibuko-Mbeje et al. (2021) [35], who demonstrated that antimycin A can be a useful model to mimic mitochondrial dysfunction and insulin resistance in cultured C2C12 myotubes. Interestingly, our study has demonstrated that such detrimental effects could be counteracted by treatment with prominent rooibos flavonoids such as aspalathin, isoorientin, and orientin in C2C12 myotubes. The current study also made use of metformin, which is a biguanide that is used as first-line treatment for patients with T2D and is also widely applied in experimental models of metabolic disease as a comparative control [36,37]. In this regard, we observed that metformin treatment improved makers of mitochondrial bioenergetics (*Ucp 2* and *Complex 1*), including the implicated transcriptional factors *(Nrf 1*, *Sirt 1*, and *Tfam*), in addition to reducing elevated ROS production in antimycin A-treated C2C12 myotubes. Besides metformin, insulin was also used as a comparative control. Our study demonstrated that insulin was effective in reverting several mitochondrial respiration markers related to antimycin A-induced ROS production and mitochondrial dysfunction. Briefly, insulin improved mitochondrial function genes (*Ucp 2*, *Complex 1*, *Nrf 1*, *Sirt 1*, and *Tfam*).

Importantly, literature entails that mitochondrial dysfunction is consistent with increased levels of ROS production within various disease conditions [6,38]. However, some dietary compounds found in rooibos have shown great potential in regulating ROS production in experimental models of mitochondrial dysfunction and metabolic disease. This statement was supported by Dludla et al., 2020 [39], who demonstrated that rooibos bioactive compounds, such as aspalathin and phenylpyruvic acid-2-*O*-*β*-D-glucoside, could reduce the excess ROS production and improve mitochondrial membrane potential in H9c2 cardiomyocytes that were exposed to high glucose concentrations mimicking the experimental model of hyperglycemia. This study also demonstrated that aspalathin, isoorientin, and orientin possess some capacity to ameliorate oxidative stress by increasing the gene expression of antioxidant genes such as *Sod 1* and *Gss* in cells treated with antimycin A. An overwhelming number of studies have indeed provided evidence that rooibos and their flavonoids have a great potential that can decrease cellular oxidative damage in various models of metabolic disease, resulting in improved intracellular antioxidant capacity [20,39,40,41]. In addition, evidence from recent research by our group indicates that aspalathin and isoorientin have the potential to reverse conditions of insulin resistance by improving energy metabolism and mitochondrial respiration [42,43]. We have observed a similar effect in this study. Here, treating skeletal muscle cells with aspalathin, isoorientin, orientin greatly improved mitochondrial basal respiration, maximal respiration, and spare capacity following exposure to antimycin A. Notably, from the literature, the decrease in mitochondrial oxidative capacity has been linked to the reduced ATP synthesis, and this complication has been observed in experimental models of T2D [44,45]. Our data showed that flavonoids aspalathin, isoorientin, and orientin could improve mitochondrial or glycolytic real-time ATP production in antimycin A-treated cells, suggesting that these bioactive compounds can enhance the overall function of the mitochondria in C2C12 skeletal muscle cells under stressful conditions. These results are of interest since clear evidence is lacking on how these flavonoids impact mitochondrial function, especially the direct effects on ATP regulation and mitochondrial bioenergetics, which is still unknown. Notably, evidence on other flavonoids such as hesperetin is reported. This compound has shown a potential to increase intracellular ATP and mitochondrial spare capacity in human primary myotubes cultured in low glucose media [46]. Thus, our results are in agreement with the current literature suggesting that naturally derived compounds can play a role in improving skeletal muscle function by enhancing ATP production and ameliorating mitochondrial dysfunction [17]. This also explains the increasing interest in understanding the therapeutic advantages of combining rooibos compounds to understand its therapeutic effects in ameliorating diverse metabolic complications [47,48]. This is an aspect that has to be further investigated to better understand the synergistic efficacy of aspalathin, isoorientin, and orientin in comparison to using each bioactive compound as a monotherapy.

**Figure 6:**
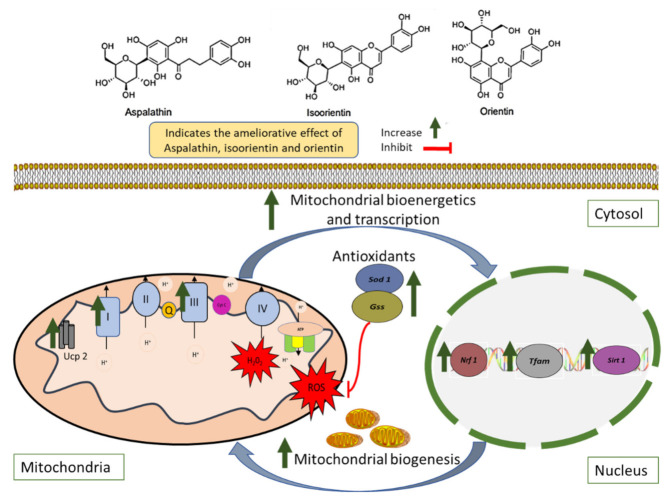
An overview of some therapeutic mechanisms linked with the ameliorative effect of rooibos flavonoids, aspalathin, isoorientin, and orientin against the complications involved in the development of mitochondrial dysfunction within the skeletal muscles. In brief, rooibos flavonoids aspalathin, isoorientin, and orientin showed the potential to improve mitochondrial bioenergetics through the upregulation of genes involved in electron transport complexes (*Complex 1* and *Complex 3*) and uncoupling protein 2 (*Ucp 2*). In partial part, due to their antioxidant properties, these flavonoids also reduced excessive reactive oxygen species (ROS) while increasing the regulation of antioxidant genes such as superoxide dismutase 1 (*Sod 1*) and glutathione synthetase (Gss). Importantly, aspalathin, isoorientin, and orientin appear effective in enhancing mitochondrial biogenesis in the skeletal muscle cells; this is evidenced by the upregulation of sirtuin 1 (*Sirt 1*) and nuclear respiratory factor 1 (*Nrf 1*), leading to the activation of mitochondrial transcription factor A (*Tfam*) and therefore enhancing mitochondrial biogenesis.

Nonetheless, existing clinical evidence already indicated that regular consumption of six cups of rooibos tea (containing relatively high levels of aspalathin, isoorientin, and orientin) per day for six weeks could lower cardiovascular disease risk by targeting the reduction in oxidative stress markers in humans [41]. In fact, previously published reviews of the literature on aspalathin, isoorientin, and orientin have highlighted the therapeutic potential of these bioactive compounds in ameliorating metabolic complications in different experimental models while also identifying necessary gaps in understanding the mechanistic insights involved [28,49,50]. However, the molecular mechanism(s) whereby flavonoids such as aspalathin, isoorientin, and orientin improve mitochondrial function in the skeletal muscle has not been elucidated. In this study, aspalathin, isoorientin, and orientin improved the expression of genes essential for an effective mitochondrial respiratory machinery and amelioration of mitochondrial ROS production, especially those that are involved in the efficient mitochondrial bioenergetics (*Ucp 2* and *Complex 1/3*) and biogenesis (*Sirt1*, *Nrf 1*, and *Tfam*) and those coding for intracellular antioxidant responses such as *Gss*, and *Sod 1* in the cultured skeletal muscle cells exposed to the detrimental effects of antimycin A (Figure 6). Even more convincingly, the current findings are consistent with our previous research [41,51], indicating that bioactive compounds found in rooibos generally show comparative effects as that of accomplished antidiabetic therapies such as metformin and insulin in ameliorating metabolic disease-associated complications in preclinical models. Nonetheless, such statements can only be confirmed through well-organized in vivo studies and potential clinical trials.

## 4. Materials and Methods

### 4.1. Materials and Reagents

Murine C2C12 skeletal muscle cells (CRL 1722) were obtained from the American Type Culture Collection (Manassas, VA, USA). Dulbecco’s modified Eagle’s medium (DMEM), Dulbecco’s phosphate-buffered saline (DPBS, pH 7.4 with calcium and magnesium), penicillin/streptomycin, and trypsin were purchased from Lonza BioWhittaker (Walkersville, MD, USA); fetal bovine serum (FBS) and horse serum (HS) were obtained from Gibco, Invitrogen (EU approved, origin: South America). Free fatty acid bovine serum albumin (BSA) was purchased from Roche (Mannheim, Germany). The 24-, and 6-well plates (Cell Bind) were purchased from The Scientific Group (Johannesburg, South Africa). Bradford kit was bought from Bio-Rad Laboratories (Hercules, CA, USA). Seahorse XF-96 microplate plates, Seahorse XF assay media, Seahorse XF base media without phenol red, and Seahorse XF-cell Mito stress, XF real-time ATP rate assay kits were purchased from Agilent (Santa Clara, CA, USA). QIAzol lysis reagent was from Qiagen (Hilden, Germany). Aspalathin (ca. 98%, Batch SZI-356-54), synthesized following an already published method by Han et al. (2014) [51], was supplied by High Force Research LTD (Durham, UK). Probes (Table 1) were purchased from Thermo Fisher Scientific (Waltham, MA, USA), whereas isoorientin (≥98.0% purity) (I1536), orientin (≥97.0% purity) (O9765), antimycin A (derived from *Streptomyces* sp.), dimethyl sulfoxide (DMSO), sodium bicarbonate (NaHCO_3_), phenol red-and glucose-free DMEM, cell culture tested water, and all other chemicals were purchased from Sigma-Aldrich (St. Louis, MO, USA).

### 4.2. Cell Culture and Differentiation

Murine C2C12 skeletal muscle cells were maintained in DMEM supplemented with 10% FBS at 37 °C in 5% CO_2_ and humidified air until they reached 80–90% confluence. Thereafter, C2C12 myotubes were seeded into 24-well plates (25,000 cells/well) for ROS production assays, and 6-well plates (75,000 cells/well) for PCR-gene expression. After 80–90% confluence, cells were maintained in a differentiation medium (DMEM supplemented with 2% (HS) for a further 3 days to facilitate myocytic differentiation. Upon differentiation, relevant assays were performed.

### 4.3. Experimental Model of Mitochondrial Dysfunction and Preparation of Treatment Compounds

To induce mitochondrial dysfunction, the C2C12 myotubes were exposed to antimycin A using a method described by Mazibuko-Mbeje et al. (2021) [35]. Briefly, antimycin A stock solution was prepared by dissolving 5 mg of antimycin A into 2.5 mL of 100% DMSO (to yield 2 mg/mL). Antimycin A-containing culture medium was prepared by diluting the antimycin A stock solution to yield a 6.25 µM working solution. Alternatively, treatment compounds were prepared according to a method described by Mazibuko, 2014 [48]. Briefly, aspalathin, isoorientin, and orientin stock solutions were prepared by dissolving the compounds in 100% DMSO to make stock solutions of 22.10 mM aspalathin and 11.5 mM isoorientin and orientin. Working solutions for aspalathin, isoorientin, and orientin (10 µM), including experimental-comparative controls, insulin (1 µM), and metformin (1 µM), were prepared by diluting the appropriate amounts of a stock solution in phenol red free DMEM (supplemented with 8 mM glucose, 3.7 g/L NaHCO_3_ and 0.1% (*w/v*) bovine serum albumin (BSA)) to yield a final working solution of DMSO < 0.01% DMSO (0.01%), as previously described [52].

### 4.4. Assessing the Production of Reactive Oxygen Species

Production of intracellular ROS was detected using an OxiSelect Intracellular ROS assay kit by making use of the DCFH-DA: dichloro-dihydro-fluoresceine diacetate dye (green fluorescence) from Cell Biolab (San Diego, CA, USA), as per manufacturer’s instruction. Briefly, skeletal muscle cells were serum starved for 30 min, exposed to antimycin A (6.25 µM) for 12 h followed by the treatment with aspalathin (10 µM), isoorientin (10 µM), orientin (10 µM), and metformin (1 µM) (comparative control) for 4 h. Insulin (1 µM) (comparative control) was added for 30 min before the termination of treatment. After the treatment, the cells were stained with 10 μM of DCFH-DA dye and incubated at 37 °C in 5% CO_2_ for 30 min. Thereafter, cells were trypsinized and collected to measure ROS production (green fluorescence) using BD Accuri C6 flow cytometer (Becton Dickinson, BD, Franklin Lakes, NJ, USA).

### 4.5. RT-PCR for mRNA Expression Analysis

The total RNA was extracted from treated C2C12 myotubes using QIAzol lysis reagent, then cleaned and reverse transcribed into complementary DNA (cDNA) using QuantiTect Reverse Transcription kit (Qiagen, Hilden, Germany), according to the manufacturer’s instructions. Gene expression was analyzed using a Quant Studio™ 7 Flex Real-Time PCR System (Thermo Scientific^TM^, MA, USA). Table 1 displays the TaqMan gene expression assays used in the study. The quantitative RT-PCR conditions were as follows: 95 °C for 10 min, followed by 40 cycles of 95 °C for 15 s and 60 °C for 1 min. Gene expression data were normalized to β2-Microglobulin.

### 4.6. Assessment of Mitochondrial Bioenergetics and Real-Time ATP Production

To assess mitochondrial bioenergetics, oxygen consumption rate (OCR) and extracellular acidification rates (ECAR) were measured with the Mito stress assay kit, while the real-time ATP assay kit was used to assess ATP synthesis. These assays were conducted using the XF 96 Extracellular Flux analyzer from Agilent (Agilent Technologies; Santa Clara, CA, USA). Briefly, C2C12 cells were seeded into 96 cell culture XF 96 microplate plate at 12,000 cells per well for 24 h, then DMEM containing 2% HS and placed in a CO_2_ incubator for another 24 h. Prior to the assay, the cells were serum starved for 30 min before mitochondrial dysfunction was induced by culturing with antimycin A (6.25 μM) for 12 h, followed by treatment with aspalathin isoorientin, and orientin for 4 h. Briefly, for both Mito stress assay and real-time ATP production, 10 μM oligomycin was injected in port A (20 μL) to inhibit ATP synthase, followed by injection of 7.5 μM carbonyl cyanide 4 trifluoromethoxy-phenylhydrazone (FCCP) in port B (22 μL) to measure maximal respiration. In both assays, a combination of antimycin A and rotenone was then added in port C (25 μL) for Mito stress assay and in port B (25 μL) for real-time assay to inhibit the activity of *Complex 1* and *Complex 3*, respectively. This was used to calculate non-mitochondrial respiration. After the assay, to control the variation between the antimycin A-treated cells and normal cells, the plates were used to quantify the total protein content using Bradford assay according to the method described by Mazibuko-Mbeje et al., 2020 [32]. Then OCR (pmol/min) was normalized relative to the protein content. OCR and ECAR were reported as absolute rates (pmoles/min/mg for OCR and mpH/min/mg for ECAR).

### 4.7. Statistical Analysis

All results are expressed as the means ± standard deviation (SD) of three independent biological experiments. The XF Mito stress test report generator and the XF real-time ATP test report generator automatically calculated the respective parameters from Wave data that was exported to Excel. The significance of difference was determined using one-way of variance (ANOVA) followed by Tukey’s post-hoc test and student *t*-test using GraphPad Prism version 8.0.1 (GraphPad Software Inc., San Diego, CA, USA). Results were considered significant at *p* < 0.05.

## 5. Conclusions

Our data demonstrated that rooibos flavonoids, aspalathin, isoorientin, and orientin improved mitochondrial function, potentiated through effective regulation of mitochondrial respiration capacity, leading to reduced production of ROS in C2C12 skeletal muscle cells under physiological conditions and in cells exposed to toxic effects of antimycin A. Although such experimental benefits are promising, the current study is with limitations, which are important to point guide future directions of research. For example, sophisticated techniques such as liquid chromatography-mass spectrometry have to be applied to determine whether these compounds reach the mitochondria and antioxidants properties of aspalathin, isoorientin, and orientin. Future studies shall also investigate the impact of these flavonoids downstream, under physiological conditions, and either as a monotherapy or in combination, on mitochondrial ROS production, and membrane depolarization. Overall, the current study showed that dietary flavonoids, aspalathin, isoorientin, and orientin, have the potential to be as effective as established pharmacological drugs such as metformin and insulin in protecting against mitochondrial dysfunction in a preclinical setting; however, such information should be confirmed in well-established in vivo disease models that are essential for further translation of results from clinical trials.

## Figures and Tables

**Table 1:** The list of TaqMan probes used in the study.

Probe	Function	Assay ID
Uncoupling Protein 2 (*Ucp 2*)	Mitochondrial Bioenergetics	Mm00627599_mL
*Complex 1*; Ubiquinol-Cytochrome c Reductase Complex Assembly Factor 1 (*Uqqc 1*)	Mitochondrial Bioenergetics	Mm00479775_mL
Complex III; Ubiquinol-Cytochrome c Reductase Complex Assembly Factor 3 (*Uqqc 3*)	Mitochondrial Bioenergetics	Mm01231041_gL
Mitochondrial Transcription Factor A (*Tfam*)	Mitochondrial Biogenesis	Mm00447485_mL
Sirtuin (*Sirt 1*)	Mitochondrial Biogenesis	Mm01168521_mL
Nuclear Respiratory Factor 1 (*Nrf 1*)	Mitochondrial Biogenesis	Mm01135606_mL
Superoxide Dismutase 1 (*Sod 1*)	Antioxidant	Mm01344233_gL
Glutathione Synthase (*Gss*)	Antioxidant	Mm00515065_mL
Beta-2-Microglobulin (*B2m*)	Housekeeping	Mm00437762_mL

## Data Availability

All data used to support the findings of this study are included within the article. Raw data can be available on request after publication.

## Sample Availability

Samples of the compounds are available through the commercial sources.

## References

[1] DeFronzo R.A., Tripathy D. (2009). Skeletal muscle insulin resistance is the primary defect in type 2 diabetes. Diabetes Care.

[2] Garneau L., Aguer C. (2019). Role of myokines in the development of skeletal muscle insulin resistance and related metabolic defects in type 2 diabetes. Diabetes Metab..

[3] Ruegsegger G.N., Creo A.L., Cortes T.M., Dasari S., Nair K.S. (2018). Altered mitochondrial function in insulin-deficient and insulin-resistant states. J. Clin. Investig..

[4] Montgomery M.K., Turner N. (2015). Mitochondrial dysfunction and insulin resistance: An update. Endocr. Connect..

[5] Zamora M. (2014). Targeting mitochondrial biogenesis to treat insulin resistance. Curr. Pharm. Des..

[6] Murphy M.P. (2013). Mitochondrial dysfunction indirectly elevates ros production by the endoplasmic reticulum. Cell Metab..

[7] Montgomery M.K. (2019). Mitochondrial dysfunction and diabetes: Is mitochondrial transfer a friend or foe?. Biology.

[8] Affourtit C. (2016). Mitochondrial involvement in skeletal muscle insulin resistance: A case of imbalanced bioenergetics. Biochim. Biophys. Acta.

[9] Fazakerley D.J., Minard A.Y., Krycer J.R., Thomas K.C., Stöckli J., Harney D.J., Burchfield J.G., Maghzal G.J., Caldwell S., Hartley R. (2018). Mitochondrial oxidative stress causes insulin resistance without disrupting oxidative phosphorylation. J. Biol. Chem..

[10] Konaté K., Yomalan K., Sytar O., Zerbo P., Brestic M., Patrick V.D., Gagniuc P., Barro N. (2014). Free radicals scavenging capacity, antidiabetic and antihypertensive activities of flavonoid-rich fractions from leaves of *Trichilia emetica* and *Opilia amentaceain* an animal model of type 2 diabetes mellitus. Evid.-Based Complement. Altern. Med..

[11] de Beer D., Malherbe C.J., Beelders T., Willenburg E.L., Brand D.J., Joubert E. (2015). Isolation of aspalathin and nothofagin from rooibos (*Aspalathus linearis*) using high-performance countercurrent chromatography: Sample loading and compound stability considerations. J. Chromatogr. A.

[12] Wang S., Liang X., Yang Q., Fu X., Rogers C.J., Zhu M., Rodgers B.D., Jiang Q., Dodson M.V., Du M. (2015). Resveratrol induces brown-like adipocyte formation in white fat through activation of AMP-activated protein kinase (AMPK) α1. Int. J. Obes..

[13] Zhang X., Jing S., Lin H., Sun W., Jiang W., Yu C., Sun J., Wang C., Chen J., Li H. (2019). Anti-fatigue effect of anwulignan via the NRF2 and PGC-1α signaling pathway in mice. Food Funct..

[14] Zare R., Nadjarzadeh A., Zarshenas M.M., Shams M., Heydari M. (2019). Efficacy of cinnamon in patients with type II diabetes mellitus: A randomized controlled clinical trial. Clin. Nutr..

[15] Babu P.V.A., Liu D., Gilbert E.R. (2013). Recent advances in understanding the anti-diabetic actions of dietary flavonoids. J. Nutr. Biochem..

[16] Arias N., Pico C., Macarulla M.T., Oliver P., Miranda J., Palou A., Portillo M.P. (2017). A combination of resveratrol and quercetin induces browning in white adipose tissue of rats fed an obesogenic diet. Obesity.

[17] Mthembu S., Dludla P., Ziqubu K., Nyambuya T., Kappo A., Madoroba E., Nyawo T., Nkambule B., Silvestri S., Muller C. (2021). The Potential role of polyphenols in modulating mitochondrial bioenergetics within the skeletal muscle: A systematic review of preclinical models. Molecules.

[18] Ku S.-K., Kwak S., Kim Y., Bae J.-S. (2014). Aspalathin and nothofagin from rooibos (*Aspalathus linearis*) inhibits high glucose-induced inflammation in vitro and in vivo. Inflammation.

[19] Mazibuko-Mbeje S.E., Dludla P.V., Roux C., Johnson R., Ghoor S., Joubert E., Louw J., Opoku A.R., Muller C.J.F. (2019). Aspalathin-enriched green rooibos extract reduces hepatic insulin resistance by modulating PI3K/AKT and AMPK Pathways. Int. J. Mol. Sci..

[20] Moens C., Bensellam M., Himpe E., Muller C.J.F., Jonas J., Bouwens L. (2020). Aspalathin protects insulin-producing β Cells against glucotoxicity and oxidative stress-induced cell death. Mol. Nutr. Food Res..

[21] von Gadow A., Joubert E., Hansmann C.F. (1997). Comparison of the antioxidant activity of aspalathin with that of other plant phenols of rooibos tea (*Aspalathus linearis*), α-Tocopherol, BHT, and BHA. J. Agric. Food Chem..

[22] Muller C.J.F., Malherbe C.J., Chellan N., Yagasaki K., Miura Y., Joubert E. (2018). Potential of rooibos, its major C-glucosyl flavonoids, andZ-2-(β-D-glucopyranosyloxy)-3-phenylpropenoic acid in prevention of metabolic syndrome. Crit. Rev. Food Sci. Nutr..

[23] Sanderson M., Mazibuko S.E., Joubert E., De Beer D., Johnson R., Pheiffer C., Louw J., Muller C. (2014). Effects of fermented rooibos (*Aspalathus linearis*) on adipocyte differentiation. Phytomedicine.

[24] Dludla P., Muller C., Louw J., Joubert E., Salie R., Opoku A., Johnson R. (2014). The cardioprotective effect of an aqueous extract of fermented rooibos (*Aspalathus linearis*) on cultured cardiomyocytes derived from diabetic rats. Phytomedicine.

[25] Huang S.-H., Kao Y.-H., Muller C.J., Joubert E., Chuu C.-P. (2020). Aspalathin-rich green *Aspalathus linearis* extract suppresses migration and invasion of human castration-resistant prostate cancer cells via inhibition of YAP signaling. Phytomedicine.

[26] Pringle N.A., Koekemoer T.C., Holzer A., Young C., Venables L., Van De Venter M. (2018). Potential therapeutic benefits of green and fermented rooibos (*Aspalathus linearis*) in dermal wound healing. Planta Med..

[27] Johnson R., De Beer D., Dludla P.V., Ferreira D., Muller C.J.F., Joubert E. (2018). Aspalathin from rooibos (*Aspalathus linearis*): A bioactive c-glucosyl dihydrochalcone with potential to target the metabolic syndrome. Planta Med..

[28] Muller C., Joubert E., de Beer D., Sanderson M., Malherbe C., Fey S., Louw J. (2012). Acute assessment of an aspalathin-enriched green rooibos (*Aspalathus linearis*) extract with hypoglycemic potential. Phytomedicine.

[29] Joubert E., De Beer D. (2011). Rooibos (*Aspalathus linearis*) beyond the farm gate: From herbal tea to potential phytopharmaceutical. S. Afr. J. Bot..

[30] Zheng H., Zhang M., Luo H., Li H. (2019). Isoorientin alleviates UVB-induced skin injury by regulating mitochondrial ROS and cellular autophagy. Biochem. Biophys. Res. Commun..

[31] Anilkumar K., Reddy G.V., Azad R., Yarla N.S., Dharmapuri G., Srivastava A., Kamal M.A., Pallu R. (2017). Evaluation of anti-inflammatory properties of isoorientin isolated from tubers of pueraria tuberosa. Oxid. Med. Cell. Longev..

[32] Mazibuko-Mbeje S.E., Ziqubu K., Dludla P.V., Tiano L., Silvestri S., Orlando P., Nyawo T.A., Louw J., Kappo A.P., Muller C.J. (2020). Isoorientin ameliorates lipid accumulation by regulating fat browning in palmitate-exposed 3T3-L1 adipocytes. Metab. Open.

[33] Sun A., Ren G., Deng C., Zhang J., Luo X., Wu X., Mani S., Dou W., Wang Z. (2016). C-glycosyl flavonoid orientin improves chemically induced inflammatory bowel disease in mice. J. Funct. Foods.

[34] Abu Bakar M.H., Cheng K.-K., Sarmidi M.R., Yaakob H., Huri H.Z. (2015). Celastrol protects against Antimycin A-induced insulin Resistance in Human Skeletal Muscle Cells. Molecules.

[35] Mazibuko-Mbeje S.E., Mthembu S.X., Dludla P.V., Madoroba E., Chellan N., Kappo A.P., Muller C.J. (2021). Antimycin A-induced mitochondrial dysfunction is consistent with impaired insulin signaling in cultured skeletal muscle cells. Toxicol. In Vitro.

[36] Sylow L., Tokarz V.L., Richter E.A., Klip A. (2021). The many actions of insulin in skeletal muscle, the paramount tissue determining glycemia. Cell Metab..

[37] Musi N., Hirshman M.F., Nygren J., Svanfeldt M., Bavenholm P., Rooyackers O., Zhou G., Williamson J.M., Ljunqvist O., Efendic S. (2002). Metformin increases AMP-activated protein kinase activity in skeletal muscle of subjects with type 2 diabetes. Diabetes.

[38] Tiwari B.S., Belenghi B., Levine A. (2002). Oxidative stress increased respiration and generation of reactive oxygen species, resulting in ATP depletion, opening of mitochondrial permeability transition, and programmed cell death. Plant Physiol..

[39] Dludla P.V., Joubert E., Muller C.J., Louw J., Johnson R. (2017). Hyperglycemia-induced oxidative stress and heart disease-cardioprotective effects of rooibos flavonoids and phenylpyruvic acid-2-O-β-D-glucoside. Nutr. Metab..

[40] Lawal A.O., Oluyede D.M., Adebimpe M.O., Olumegbon L.T., Awolaja O.O., Elekofehinti O.O., Crown O.O. (2019). The cardiovascular protective effects of rooibos (*Aspalathus linearis*) extract on diesel exhaust particles induced inflammation and oxidative stress involve NF-κB- and Nrf2-dependent pathways modulation. Heliyon.

[41] Marnewick J.L., Rautenbach F., Venter I., Neethling H., Blackhurst D.M., Wolmarans P., Macharia M. (2011). Effects of rooibos (*Aspalathus linearis*) on oxidative stress and biochemical parameters in adults at risk for cardiovascular disease. J. Ethnopharmacol..

[42] Mazibuko-Mbeje S.E., Dludla P.V., Johnson R., Joubert E., Louw J., Ziqubu K., Tiano L., Silvestri S., Orlando P., Opoku A.R. (2019). Aspalathin, a natural product with the potential to reverse hepatic insulin resistance by improving energy metabolism and mitochondrial respiration. PLoS ONE.

[43] Ziqubu K., Muller C.J.F., Dludla P.V., Mthembu S.X.H., Obonye N., Louw J., Kappo A.P., Silvestri S., Orlando P., Tiano L. (2020). Impact of isoorientin on metabolic activity and lipid accumulation in differentiated adipocytes. Molecules.

[44] Ye X., Shen Y., Ni C., Ye J., Xin Y., Zhang W., Ren Y. (2019). Irisin reverses insulin resistance in C2C12 cells via the p38-MAPK-PGC-1α pathway. Peptides.

[45] Dickerson R., Banerjee J., Rauckhorst A., Pfeiffer U.R., Gordillo G.M., Khanna S., Osei K., Roy S. (2015). Does oral supplementation of a fermented papaya preparation correct respiratory burst function of innate immune cells in type 2 diabetes mellitus patients?. Antioxidants Redox Signal..

[46] Biesemann N., Ried J.S., Ding-Pfennigdorff D., Dietrich A., Rudolph C., Hahn S., Hennerici W., Asbrand C., Leeuw T., Strübing C. (2018). High throughput screening of mitochondrial bioenergetics in human differentiated myotubes identifies novel enhancers of muscle performance in aged mice. Sci. Rep..

[47] Dludla P.V., Muller C.J.F., Louw J., Mazibuko-Mbeje S.E., Tiano L., Silvestri S., Orlando P., Marcheggiani F., Cirilli I., Chellan N. (2020). The combination effect of aspalathin and phenylpyruvic acid-2-O-β-d-glucoside from rooibos against hyperglycemia-induced cardiac damage: An in vitro study. Nutrients.

[48] Mazibuko S.E. (2014). In Vitro and In Vivo Effect of Aspalathus Linearis and Its Major Polyphenols on Carbohydrate and Lipid Metabolism in Insulin Resistant Models. http://hdl.handle.net/10530/1319.

[49] Ziqubu K., Dludla P.V., Joubert E., Muller C.J., Louw J., Tiano L., Nkambule B.B., Kappo A.P., Mazibuko-Mbeje S.E. (2020). Isoorientin: A dietary flavone with the potential to ameliorate diverse metabolic complications. Pharmacol. Res..

[50] Lam K.Y., Ling A.P.K., Koh R.Y., Wong Y.P., Say Y.-H. (2016). A review on medicinal properties of orientin. Adv. Pharmacol. Sci..

[51] Han Z., Achilonu M.C., Kendrekar P.S., Joubert E., Ferreira D., Bonnet S.L., Van Der Westhuizen J.H. (2013). Concise and scalable synthesis of aspalathin, a powerful plasma sugar-lowering natural product. J. Nat. Prod..

[52] Dludla P.V., Jack B., Viraragavan A., Pheiffer C., Johnson R., Louw J., Muller C.J. (2018). A dose-dependent effect of dimethyl sulfoxide on lipid content, cell viability and oxidative stress in 3T3-L1 adipocytes. Toxicol. Rep..

